# Effective Sensor Selection and Data Anomaly Detection for Condition Monitoring of Aircraft Engines

**DOI:** 10.3390/s16050623

**Published:** 2016-04-29

**Authors:** Liansheng Liu, Datong Liu, Yujie Zhang, Yu Peng

**Affiliations:** Department of Automation Measurement and Control, Harbin Institute of Technology, Harbin 150001, China; lianshengliu@hit.edu.cn (L.L.); liudatong@hit.edu.cn (D.L.); hnhyzyjlh@hit.edu.cn (Y.Z.)

**Keywords:** condition monitoring, sensor selection, anomaly detection, mutual information, Gaussian Process Regression

## Abstract

In a complex system, condition monitoring (CM) can collect the system working status. The condition is mainly sensed by the pre-deployed sensors in/on the system. Most existing works study how to utilize the condition information to predict the upcoming anomalies, faults, or failures. There is also some research which focuses on the faults or anomalies of the sensing element (*i.e.*, sensor) to enhance the system reliability. However, existing approaches ignore the correlation between sensor selecting strategy and data anomaly detection, which can also improve the system reliability. To address this issue, we study a new scheme which includes sensor selection strategy and data anomaly detection by utilizing information theory and Gaussian Process Regression (GPR). The sensors that are more appropriate for the system CM are first selected. Then, mutual information is utilized to weight the correlation among different sensors. The anomaly detection is carried out by using the correlation of sensor data. The sensor data sets that are utilized to carry out the evaluation are provided by National Aeronautics and Space Administration (NASA) Ames Research Center and have been used as Prognostics and Health Management (PHM) challenge data in 2008. By comparing the two different sensor selection strategies, the effectiveness of selection method on data anomaly detection is proved.

## 1. Introduction

In modern industry, systems are becoming more and more complex, especially for the machine system. For example, an aircraft consists of several subsystems and millions of parts [1]. To enhance its reliability, the condition of the main subsystems should be monitored. As the aircraft’s heart, the condition of the engine directly affects its operation and safety. The engine works in a very harsh environment (e.g., high pressure, high temperature, high rotation speed, *etc.*). Therefore, its condition should be monitored thoroughly. The situation in other complex engineering systems is similar [2]. One effective strategy to enhance the reliability of the system is to utilize Condition Monitoring (CM).

For improving the availability of equipment, many mathematical models and methodologies have been developed to realize and enhance the performance of CM. For example, dynamic fault tree is proposed to filter false warnings of the helicopter. The methodology is based on operational data analysis and can help identify the abnormal events of the helicopter [3,4]. For power conversion system CM, it is important to construct the measurement of damage indicators to estimate the current aging status of the power device, which include threshold voltage, gate leak current, *etc.* [5]. The optimum type, number, and location of sensors for improving fault diagnosis is realized in [6]. Gamma process has been successfully applied to describe a certain type of degradation process [7,8]. CM can also be utilized to monitor the system’s sudden failure, which is carried out by forming an appropriate evolution progress [9,10].

In addition, CM can help provide the scheduled maintenance, reduce life-cycle costs, *etc.* [11]. Hence, it is important to sense the condition of the engine. Many existing research works have been carried out to realize CM. Among the available methods, one of the most promising technologies is Prognostics and Health Management (PHM). PHM has been applied in industrial systems [12,13] and avionics systems [14,15]. For the aircraft engine, PHM can provide failure warnings, extend the system life, *etc.* [16].

In summary, PHM methods can be classified into three major categories: model-based method, experience-based method, and data-driven method [17]. If the system can be represented by an exact model, the model-based method is applicable [18]. However, it is difficult for the accurate model to be identified in many practical applications. Hence, the model-based method is difficult to be put into use for complex systems [19]. For the experience-based approach, the stochastic model is necessary, which is often not accurate for complex systems [20]. Compared with the model-based method and the experience-based method, the data-driven method utilizes the direct data collected by instruments (most are based on sensors) and has become the primary selection for complex systems [21,22]. Many sensors are deployed on or inside the engine to sense various physical parameters (e.g., operation temperature, oil temperature, vibration, pressure, *etc.*) [23]. The operational, environmental and working conditions of the aircraft engine can be monitored by utilizing these sensors.

The aim of CM is to identify the unexpected anomalies, faults, and failures of the system [24]. In theory, more sensor data are more helpful for CM. However, too many sensors will bring a large amount of data processing, system costs, *etc.* [11]. Therefore, one typical strategy is to select some sensors which can provide better CM results. One common method is to observe the degradation trend of sensor data [25,26]. Then, the appropriate sensors will be selected for CM. In our previous work [27], one metric based on information theory for sensor selection has been proposed. This article is the extension of our previous work [27] and aims at discovering the correlation between sensor selection strategy and data anomaly detection. Reasonable sensor selection can be considered as how to choose data for CM. The correctness of sensed data is significant for the system CM. The influence of sensor selection strategy on data anomaly detection is studied in this article. In this way, the correctness of condition data can help enhance the result of fault diagnosis and failure prognosis. Much work has been carried out for data anomaly detection [28,29,30]. However, to the best of our knowledge, there is no work that considers the influence of sensor selection strategy on data anomaly detection.

To prove the correlation between sensor selection strategy and data anomaly detection, we first select the sensors that are more suitable for system CM. The methodology is based on information theory and the details can be found in our previous work [27]. Then, mutual information is utilized to weight the dependency among sensors. In the domain of probability theory, mutual information is one type of method for correlation analysis and is an effective tool to measure dependency between random variables [31]. It complies with the motivation of our study. To prove the influence of sensor selection strategy on data anomaly detection, mutual information is utilized to find the target sensor and the training sensor.

Then, the classical Gaussian Process Regression (GPR) is adopted to detect the target sensor data anomalies [32]. The parameters of GPR are calculated by the training sensor data. The target sensor data is detected by the trained GPR. For evaluation, the sensor data sets that are provided by the National Aeronautics and Space Administration (NASA) Ames Research Center for aircraft engine CM are utilized. The experimental results show the effectiveness of reasonable sensor selection on data anomaly detection. The claimed correlation between sensor selection strategy and data anomaly detection is one typical problem in the engineering system. The insights founded by the proposed method are expected to help provide more reasonable CM for the system.

The rest of this article is organized as follows. Section 2 introduces the aircraft engine which is utilized as the CM target. Section 3 presents the related theories, including information theory, GPR, and anomaly detection metrics. Section 4 illustrates the detailed evaluation results and analysis. Section 5 concludes this article and points out the future works.

## 2. Aircraft Engine for Condition Monitoring

**Figure 1:**
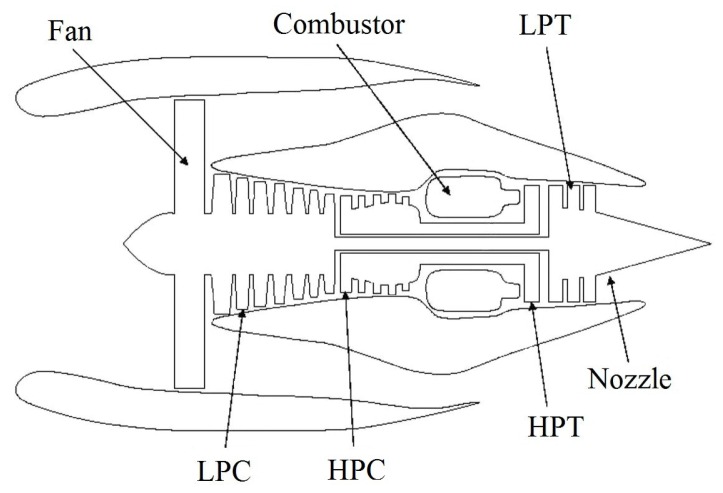
Diagram for the aircraft gas turbine engine [33].

The turbofan aircraft engine is utilized as the objective system in this study. An important requirement for an aircraft engine is that its working condition can be sensed correctly. Then, some classical methodologies can be adopted to predict the upcoming anomalies, faults or failures. The typical architecture of the engine is shown in Figure 1 [33]. The engine consists of Fan, Low-Pressure Compressor (LPC), High-Pressure Compressor (HPC), Combustor, High-Pressure Turbine (HPT), Low-Pressure Turbine (LPT), Nozzle, *etc*.

**Figure 2:**
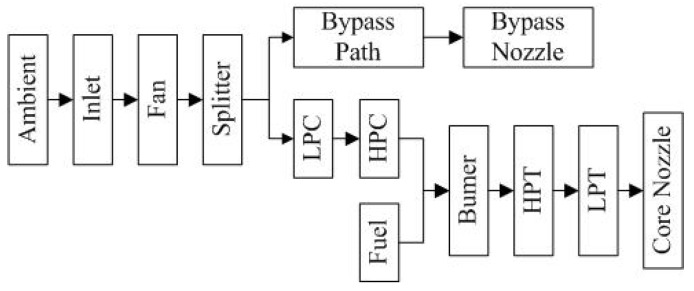
Layout of various modules and their connections [33].

The engine illustrated in Figure 1 is simulated by C-MAPSS (Commercial Modular Aero-Propulsion System Simulation). C-MAPSS is a simulating tool and has successfully been utilized to imitate the realistic work process of a commercial turbofan engine. Regarding operational profiles, a number of input parameters can be edited to realize expected functions. Figure 2 shows the routine assembled in the engine.

The engine has a built-in control system, which includes a fan-speed controller, several regulators and limiters. The limiters include three high-limit regulators that are used to prevent the engine from exceeding the operating limits. These limits mainly include core speed, engine-pressure ratio, and HPT exit temperature. The function of regulators is to prevent the pressure going too low. These situations in C-MAPSS are the same as the real engine.

The aircraft engine directly influences the reliability and the safety of the aircraft. Therefore, the unexpected conditions of the engine should be monitored. The reliability can be understood from three factors. First, the failure of the main components (LPC, HPC, Combustor, *etc.*) can lead to the failure of the aircraft engine. Secondly, if the information transmitted to actuators is faulty, it will cause the failure of the aircraft engine. Finally, the external interferences (e.g., birds striking) can result in the failure of the aircraft engine.

In this article, the condition data of the aircraft engine are the concern and the anomaly detection of the condition data is the focus. To monitor the condition of the engine, several types of physical parameters can be utilized, such as temperature, pressure, fan speed, core speed, air ratio, *etc*. A total of 21 sensors are installed on or inside different components of the aircraft engine to collect its working conditions, as illustrated in Table 1. The deterioration and faults of the engine can be detected by analyzing these sensors data [34].

## 3. Related Theories

In this section, the related theories utilized in this study are introduced, including information theory (entropy, permutation entropy, and mutual information), Gaussian Process Regression and anomaly detection metrics.

### 3.1. Information Theory

#### 3.1.1. Entropy

Every sensor $S_{x_{i}}$ (i=1,...,n) deployed in the location $l_{i}$ to sense the target condition variable $x_{i}$ can be be regarded as a random variable $X_{S_{i}}$. The acquisition result can be expressed by time series data {yi(t), yi(t+1),…,yi(T)}. The data can also be visualized as a realization of $X_{S_{i}}$ on the time window [*t*,*T*]. Sensor data sets can be described by the probability distribution. The information contained in the data can be measured by entropy, which is defined by Equation (1) [35].

(1) $$H=-\sum_{i=1}^{N}p_{i}\left(x\right)logp_{i}\left(x\right)$$

where $p_{i}\left(x\right)$ indicates the probability of the *i*th state, and *N* denotes the total number of states that the process of $X_{S_{i}}$ exhibits.

For a continuous random variable *X*, the probability is expressed by the probability density function f(x) and the entropy is defined as

(2) $$H=-\int_{S}f\left(x\right)logf\left(x\right)dx$$

where *S* is the set of the random variables.

If the base of the logarithm is 2, the entropy is measured in bits. If the logarithm is based on *e*, the entropy is measured in nats. Besides the base of 2 and *e*, the logarithm can be based on other dimensions, and the definition entropy can be changed for different applications. To be simple, the base of the logarithm in our study is based on 2, and the entropy will be measured in bits.

To help understand the entropy, a simple example is given as follows. Let

(3) $$X=\left\{\begin{array}{cc} 1, & p\\ 0, & 1-p \end{array}\right.$$

Then

(4) H=−plogp−(1−p)log(1−p)

**Figure 3:**
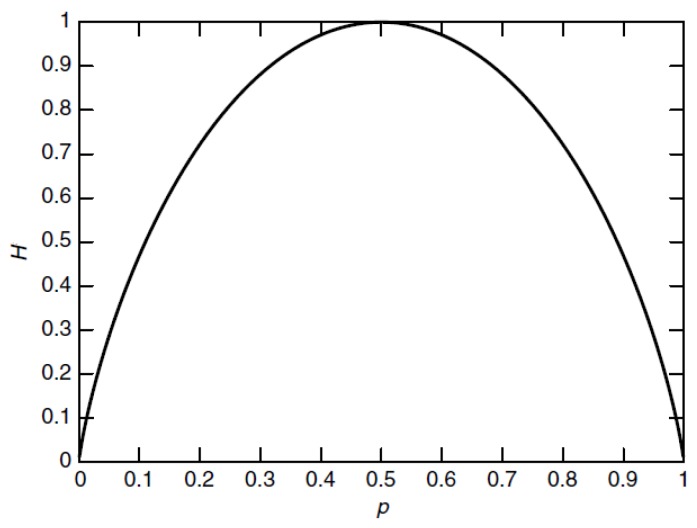
Entropy *vs.* probability.

The graph of the entropy in Equation (4) is described in Figure 3. Some basic properties of entropy can be drawn from Figure 3. It is concave and the value of entropy is 0 when p=0 or 1. When the probability is 0 or 1, it means that the variable is not random and there is no uncertainty. Hence, the information contained in the data set is 0. On the other hand, the uncertainty is maximum when p=1/2 , which corresponds to the maximum entropy value.

Entropy can be applied to measure the information contained in the sensor data. For CM, the data that have the characteristics of degradation trend are more suitable. In the following subsection, the permutation entropy that is utilized to calculate the degradation trend of the sensor data will be illustrated.

#### 3.1.2. Permutation Entropy

The sensor data {yi(t), yi(t+1),…,yi(T)} includes T! permutation of possible ranks. The frequency of each permutation T! can be calculated by

(5) $$p\left(\pi \right)=\frac{t|0⩽t⩽T-n,(x_{t+1},\cdots ,x_{t+n})\;has\;type\;\pi }{T-n+1}$$

The permutation entropy of order *n*≥2 is defined as

(6) H(n)=−∑p(π))logp(π))

The permutation entropy reflects the information contained in comparing *n* consecutive values of the sensor data. It is clear that

(7) 0⩽H(n)⩽logn!

where the lower bound is attained for an increasing or decreasing data set.

The permutation entropy of order *n* divided by n−1

(8) h(n)=H(n)/(n−1)

can be made use of for determining some properties of dynamics. The information contained in sorting *n*th value is among the previous n−1 permutation entropy.

The increasing or decreasing trend of sensor data set can be represented by 2! permutation entropy which can be calculated by

(9) H(2)=−plogp−(1−p)log(1−p)

where *p* denotes the increasing or decreasing probability of order n=2. If *p* indicates the increasing probability, then 1−p is the decreasing probability.

#### 3.1.3. Mutual Information

In order to measure the conditional entropy of one random variable on the other random variable, the conditional entropy H(Y|X) can be adopted, which is defined by

(10) $$H\left(Y|X\right)=\sum_{i=1}^{n}p\left(x_{i}\right)H\left(Y|X=x_{i}\right)=-\sum_{i=1}^{n}\sum_{j=1}^{m}p\left(x_{i},y_{j}\right)logp\left(y_{j}|x_{i}\right)$$

For two random variables *X* and *Y*, the mutual information I(Y;X) is the reduction between the two random variables and can be calculated by

(11) $$I\left(Y;X\right)=H\left(Y\right)-H\left(Y|X\right)=\sum_{x,y}p\left(x,y\right)log\left(p\left(x,y\right)/p\left(x\right)p\left(y\right)\right)$$

### 3.2. Gaussian Process Regression

Gaussian Process (GP) is the generalization of Gaussian distribution and is one type of important stochastic process [32,36]. The parameters are not required when the GP is modeled. Based on the input data sets, the corresponding functions {$f\left(x_{1}\right),\cdots ,f\left(x_{n}\right)$} comprise a collection of random variables $D={\left\{x_{n}\right\}}_{n=1}^{N},x\in R^{d}$ which obey the joint Gaussian distribution. The functions {$f\left(x_{1}\right),\cdots ,f\left(x_{n}\right)$} can be used to form GP, as given by

(12) $$f\left(x\right)∼GP(m\left(x\right),k\left(x_{i},x_{j}\right))$$

(13) m(x)=E[f(x)]

(14) $$k\left(x_{i},x_{j}\right)=E\left[\left(f\left(x_{i}\right)-m\left(x_{j}\right)\right)\left(f\left(x_{i}\right)-m\left(x_{j}\right)\right)\right]$$

where m(x) denotes the mean function and $k(x_{i},x_{j})$ indicates the covariance function.

In practical scenarios, the function values contain noise which can be expressed by

(15) y=f(x)+ε

where *ε* is the white noise and $\varepsilon \in N(0,\sigma_{n}^{2})$. In addition, *ε* is independent on f(x). Moreover, if f(x) is used to formulate GP, the observation *y* is also GP, which can be represented by

(16) $$y∼GP(m\left(x\right),k\left(x_{i},x_{j}+\sigma_{n}^{2}\delta_{ij}\right)$$

where $\delta_{ij}$ is the Dirac function. When the value of *i* and *j* is equivalent, the value of $\delta_{ij}$ is 1.

GPR is one type of probability technology for the regression problem and is restricted by the prior distribution. By utilizing the available training data sets, the estimation of the posterior distribution can be obtained. Hence, this methodology makes use of the functional space defined by the prior distribution of GP. The prediction output of GP function of the posterior distribution can be calculated by the Bayesian framework [37].

In the assumption, the data sets of $D_{1}={\left\{x_{i},y_{i}\right\}}_{i=1}^{N}$ are the training data sets and $D_{2}={\left\{x_{i}^{*},y_{i}\right\}}_{i=1}^{N^{*}}$ are the testing data sets, $x_{i},x_{i}^{*}\in R^{d}$ and *d* is the input dimension, *m* and $m^{*}$ are the mean vector of the training data sets and the testing data sets respectively. $f(x_{*})$ is the function output with test input, which complies with the vector $f_{*}$, and *y* is the training vector. According to Equation (15), $f_{*}$ and *y* comply with the joint Gaussian distribution, as illustrated by

(17) $$\left(\begin{array}{c} y\\ f_{*} \end{array}\right)∼\left(\left[\begin{array}{c} m\\ m_{*} \end{array}\right],\left(\begin{array}{cc} C(X,X) & K(X,X_{*})\\ K(X_{*},X) & K(X_{*},X) \end{array}\right)\right)$$

where $C\left(X,X\right)=K\left(X,X\right)+\delta_{ij}I$ is the covariance matrix of the training data sets, $\delta_{ij}$ refers to the variance of the white noise, $I\in R^{N\times N}$ indicates the unit matrix, $K\left(X,X_{*}\right)\in R^{N\times N^{*}}$ denotes the covariance matrix for the training data sets and the testing data sets, and $K(X_{*},X_{*})$ refers to the covariance of the testing data sets.

According to the characteristics of GP, the posterior conditional distribution of $f_{*}$ can be achieved by

(18) $$f_{*}|X,y,X_{*}∼N\left(\bar{f_{*}},cov\left(f_{*}\right)\right)$$

(19) $$\bar{f_{*}}=E\left[f_{*}|X,y,X_{*}\right]=m+K\left(X_{*},X\right)C{\left(X,X\right)}^{-1}\left(y-m\right)$$

(20) $$cov\left(f_{*}\right)=K\left(X_{*},X_{*}\right)-C{\left(X,X\right)}^{-1}K\left(X,X_{*}\right)$$

### 3.3. Anomaly Detection Metrics

Three metrics are usually utilized to measure the accuracy of anomaly detection, which include *F*alse *P*ositive *R*atio (FPR), *F*alse *N*egative *R*atio (FNR) and Accuracy (ACC). FPR is the ratio that the anomalous data are falsely detected, which can be calculated by

(21) $$FPR=\frac{FN}{TP+FN}\times 100\%$$

where FN is the amount of normal data identified as the anomalous data, TP+FN is the sum of the normal data.

FNR is the ratio that the anomalous data are detected in error and accepted, which can be calculated by

(22) $$FNR=\frac{FP}{FP+TN}\times 100\%$$

where FP is the amount of anomalous data identified as normal data, FP+TN is the sum of the anomalous data. The smaller values of FNR and FPR mean that the performance of the anomaly detection method is better.

ACC is the ratio that the anomalous data are detected in error and accepted, which can be calculated by

(23) $$ACC=\frac{TP+TN}{FP+FN+TN+TP}\times 100\%$$

where TP+TN is the amount of the anomalous data detected as anomaly and the normal data identified as positive data, FP+FN+TN+TP is the number of all data detected.

## 4. Experimental Results and Analysis

**Figure 4:**
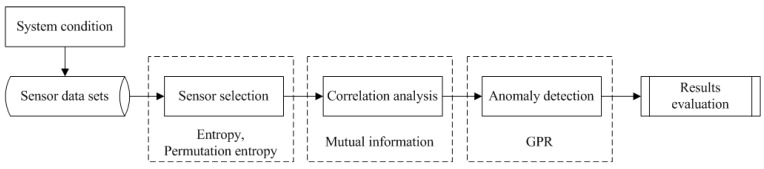
Framework of sensor selection and data anomaly detection.

In this section, we first present the overview of the sensor data for CM of the aircraft engine. The suitable sensors for CM are first selected. Then, the most related sensors are carried out the following data anomaly detection. The framework of sensor selection and data anomaly detection is shown in Figure 4.

After the system condition collected by pre-deployed sensors, the sensor data sets can be utilized for the following analysis. The sensor selection strategy for CM is based on our previous work. Then, mutual information among sensors is calculated to weight the correlation. The target sensor that will be used for data anomaly detection should be same for the following comparison of detection performance analysis. The sensor that has the largest value of mutual information to the target sensor is used to train GPR. By analyzing the anomaly detection results of the target sensor, the influence of sensor selection strategy on data anomaly detection is proved.

### 4.1. Sensor Data Description

As introduced in Section 2, there are 21 sensors that are utilized to sense the engine condition. The experiments are carried out under four different combinations of operational conditions and failure modes [34]. The sensor data sets of the overall experiments are illustrated in Table 2.

Each data set is divided into the training and testing subsets. The training set contains run-to-failure information, while the testing set has up-to-date data. In order to evaluate the effectiveness of our method, the data set 1 which has one fault mode (HPC degradation) and one operation condition (Sea Level) is picked first, as shown in Table 3.

### 4.2. Sensor Selection Procedure

The sensor selection procedure method is based on our previous work [27], which is based on the quantitative sensor selection strategy. The procedure includes two steps. First, the information contained in the sensor data is weighted by entropy, as introduced in Section 3.1.1. Then, the modified permutation entropy is calculated, which only considers the 2! permutation entropy value. The 2! permutation entropy value can be utilized to describe the increasing or decreasing trend of the sensor data. In this way, the sensors which are more suitable for CM will be selected.

The quantitative sensor selection strategy aims at finding out the information contained in the sensor data sets. The output of every sensor can be considered as one random variable. To measure the information contained in the sensor data, the entropy which calculates the probability of every data is utilized. The larger value of entropy means that the data contains more information, as introduced in Section 3.1. Then, the suitable sensors for system CM are selected by utilizing the improved permutation entropy that considers the probability of the increasing or decreasing of the two adjacent sensor data. This feature can be utilized to describe the increasing or decreasing trend of the sensor data and is preferred for system CM.

The work in [25] utilizes the observing sensor selection strategy and selects seven sensors for the aircraft engine CM. The observing method is based on subjective judgement. To improve the effectiveness of the quantitative sensor selection strategy, the number of sensors selected in [27] is the same as [25]. In this study, we also adopt the same data set and the same seven sensors as in our previous work [27] which are #3, #4, #8, #9, #14, #15, and #17. The sensors selected in [25] are #2, #4, #7, #8, #11, #12, and #15. In the following evaluation, the experiments are carried out between these two groups of sensors to prove the merit of the quantitative sensor selection strategy.

### 4.3. Data Anomaly Detection and Analysis

In order to evaluate the effectiveness of sensor selection strategy on data anomaly detection, we first calculate the mutual information among the sensors in the two groups, respectively. Mutual information can be utilized to weight the correlation among sensors.

Mutual information values among the sensors selected by the quantitative sensor selection strategy for data set 1 are shown in Table 4.

Mutual information values among the sensors selected by the observing sensor selection strategy for data set 1 are shown in Table 5.

To compare the effectiveness of the quantitative sensor selection method with the observing sensor selection method, the testing sensor data should be same and the training sensor data should be different. By analyzing illustrated sensors in Table 4 and Table 5, sensor #15 is selected as the target testing sensor. For the quantitative sensor selection, sensor #3 is chosen to be the training sensor. For the observing sensor selection, sensor #2 is chosen to be the training sensor.

**Figure 5:**
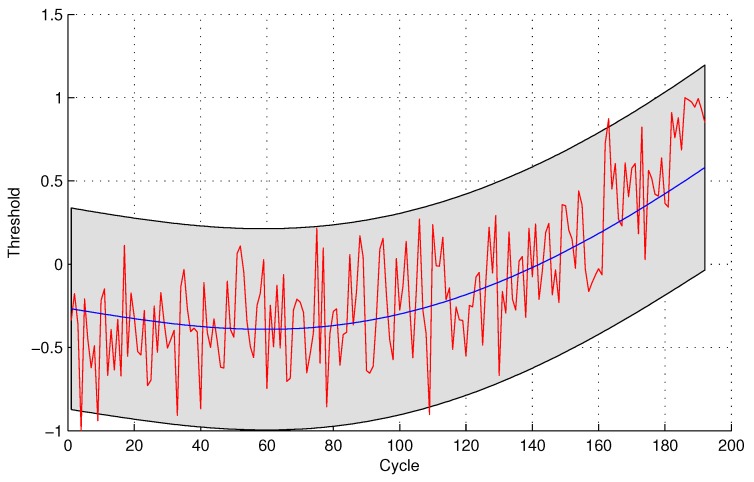
Anomaly detection for sensor data set 1 with Gaussian Process Regression (GPR) for the quantitative sensor selection.

**Figure 6:**
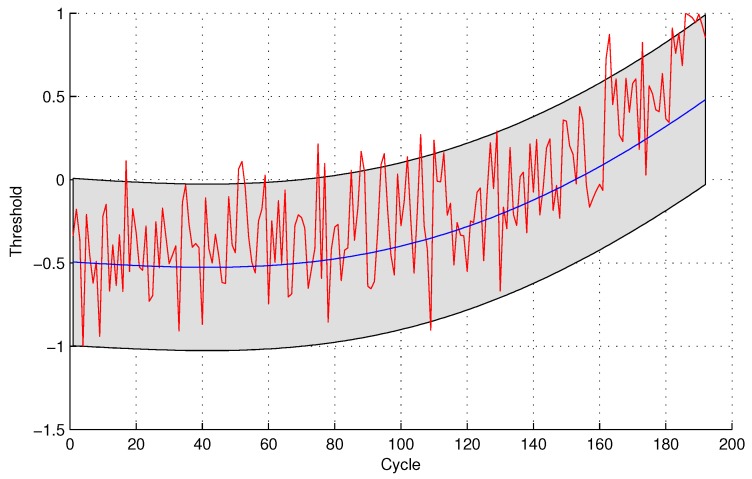
Anomaly detection for sensor data set 1 with GPR for the observing sensor selection.

In the following evaluation step, GPR is utilized to detect the testing sensor data. The parameters of GPR are trained by sensor #4 and sensor #3 for the two sensor selection strategies, respectively, due to the maximal mutual information with the same sensor. The experimental results of data anomaly detection for data set 1 are shown in the following Figure 5 and Figure 6.

The number of normal sensor data detected as anomalous data is four and 24 for the two methods, respectively. For the quantitative sensor selection strategy, the FPR of data anomaly detection is

$$FPR=\frac{4}{4+188}\times 100\%=2.08\%$$

For the observing sensor selection strategy, the FPR of data anomaly detection is

$$FPR=\frac{24}{24+168}\times 100\%=12.50\%$$

Another sensor data set is also carried out to evaluate the proposed method. Mutual information values among the sensors selected by the quantitative sensor selection strategy for data set 2 are shown in Table 6.

Mutual information values among the sensors selected by the observing sensor selection strategy for data set 2 are shown in Table 7.

**Figure 7:**
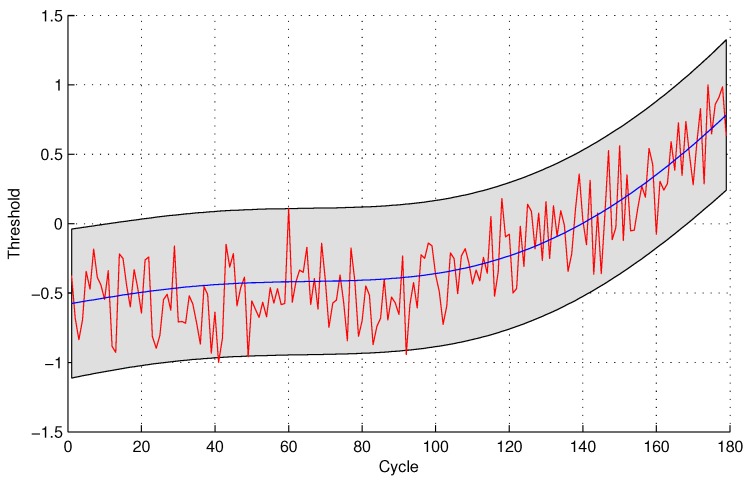
Anomaly detection for sensor data set 2 with GPR for the quantitative sensor selection.

**Figure 8:**
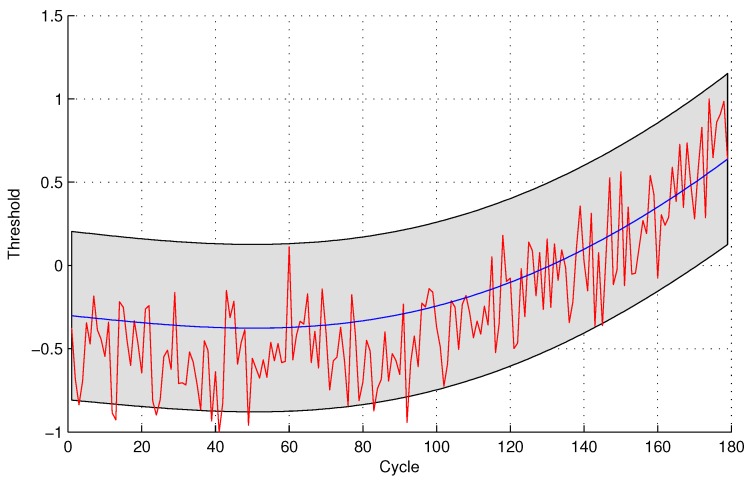
Anomaly detection for sensor data set 2 with GPR for the observing sensor selection.

The experimental results of data anomaly detection for data set 2 are shown in the following Figure 7 and Figure 8.

For the quantitative sensor selection strategy, the FPR of data anomaly detection is

$$FPR=\frac{4}{4+175}\times 100\%=2.23\%$$

For the observing sensor selection strategy, the FPR of data anomaly detection is

$$FPR=\frac{10}{10+169}\times 100\%=5.59\%$$

To evaluate the effectiveness in further, two additional data sets of another working condition are utilized to carry out the following experiments. For the third data set, mutual information values among the sensors selected by the quantitative sensor selection strategy are shown in Table 8.

Mutual information values among the sensors selected by the observing sensor selection strategy for data set 3 are shown in Table 9.

**Figure 9:**
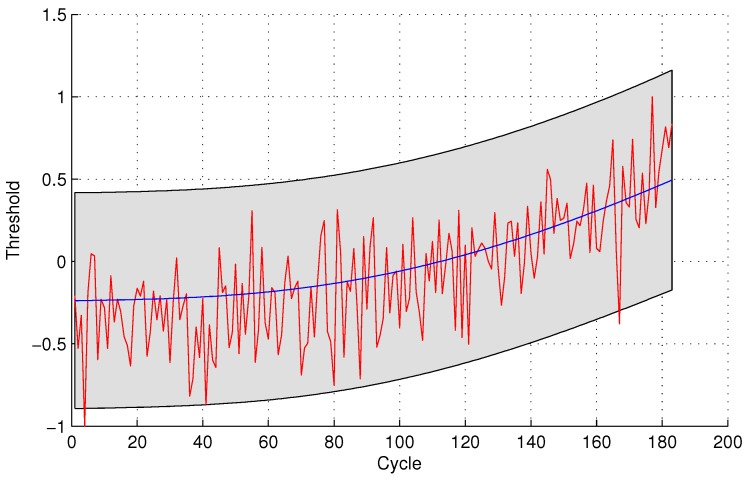
Anomaly detection for sensor data set 3 with GPR for the quantitative sensor selection.

**Figure 10:**
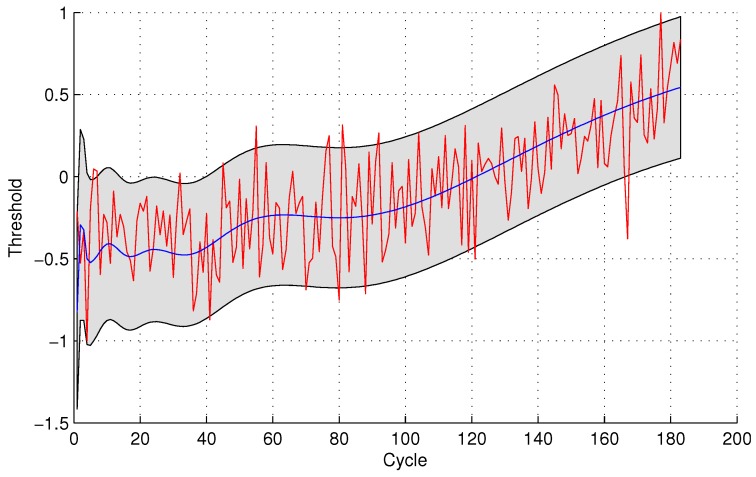
Anomaly detection for sensor data set 3 with GPR for the observing sensor selection.

The experimental results of data anomaly detection for data set 3 are shown in the following Figure 9 and Figure 10.

For the quantitative sensor selection strategy, the FPR of data anomaly detection is

$$FPR=\frac{3}{3+180}\times 100\%=1.64\%$$

For the observing sensor selection strategy, the FPR of data anomaly detection is

$$FPR=\frac{17}{17+166}\times 100\%=9.29\%$$

For the fourth data set, mutual information values among the sensors selected by the quantitative sensor selection strategy are shown in Table 10.

Mutual information values among the sensors selected by the observing sensor selection strategy for data set 4 are shown in Table 11.

**Figure 11:**
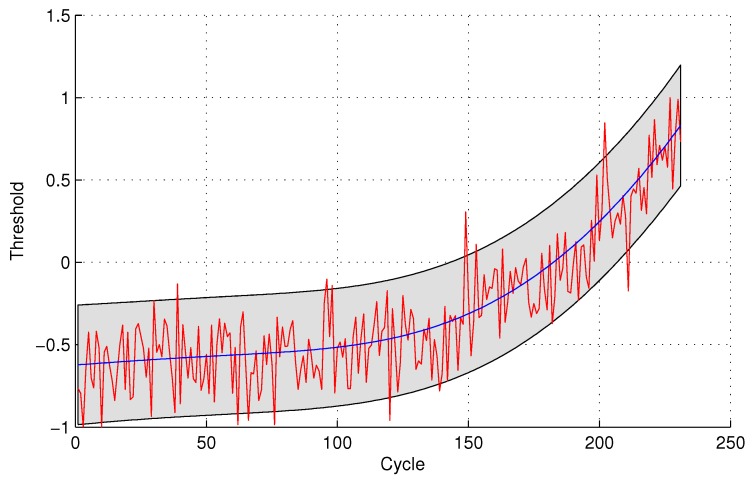
Anomaly detection for sensor data set 4 with GPR for the quantitative sensor selection.

**Figure 12:**
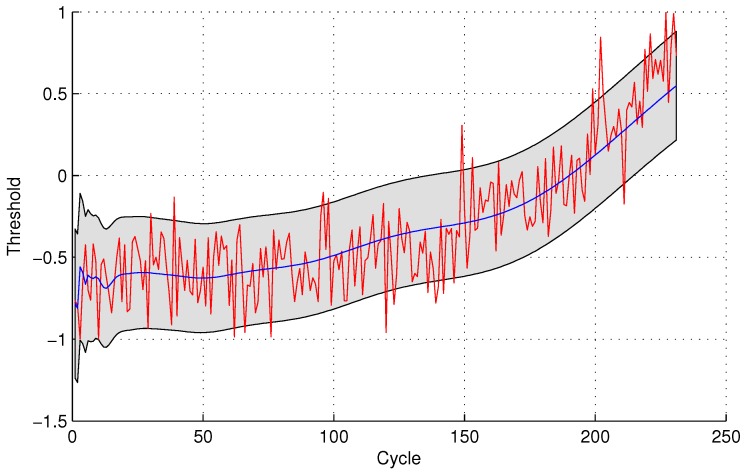
Anomaly detection for sensor data set 4 with GPR for the observing sensor selection.

The experimental results of data anomaly detection for data set 4 are shown in the following Figure 11 and Figure 12.

For the quantitative sensor selection strategy, the FPR of data anomaly detection is

$$FPR=\frac{18}{18+213}\times 100\%=7.79\%$$

For the observing sensor selection strategy, the FPR of data anomaly detection is

$$FPR=\frac{25}{25+206}\times 100\%=10.82\%$$

For the quantitative sensor selection strategy, the four values of FPR are 2.08%, 2.23%, 1.64%, and 7.79%, respectively. For the observing sensor selection strategy, the four values of FPR are 12.50%, 5.59%, 9.29%, and 10.82%, respectively. In the above mentioned data sets, four pairs of sensor data are selected randomly to implement the data anomaly detection. The values of FPR are 32.81%, 50.84%, 54.10%, and 25.97%, respectively. To compare the performance of three types of data anomaly detection, the mean and standard deviation of FPR are calculated, as illustrated in Table 12.

From the evaluation experimental results, it can be seen that the quantitative sensor selection not only has better FPR values but also the mean and the standard deviation. Therefore, compared with the observing sensor selection strategy and random sensor selection, the performance of the quantitative sensor selection strategy has better performance on data anomaly detection at a certain degree.

Compared with the observing sensor selection strategy and the random sensor selection, the quantitative sensor selection strategy achieves smaller numerical values of FPR. The mean value and the standard deviation value of the quantitative sensor selection strategy are also smaller than the other two methods. The performance of the sensor selection strategy on data anomaly detection is validated at a certain degree. However, the effectiveness needs to be validated further with the involvement of more data, especially the anomalous data.

## 5. Conclusions

In this article, we present one typical problem in engineering, which is how to select sensors for CM. Compared with the observing sensor selection method, the quantitative sensor selection method is more suitable for system CM and data anomaly detection. The effectiveness is expected to enhance the reliability and performance of system CM. In this way, it can guarantee that the basic sensing information is correct. Therefore, the system reliability can be enhanced further. The method which can be utilized for selecting sensors to carry out anomaly detection is also illustrated. Experimental results with the sensor data sets that are obtained from aircraft engine CM show the correlation between sensor selection strategy and data anomaly detection.

In future work, we will focus on how to utilize multidimensional data sets to carry out anomaly detection. The anomaly detection accuracy is expected to be further improved. Then, the computing resource will be considered, especially for the online anomaly detection and the limited computing resource scenario. The uncertainty about anomaly detection results will also be taken into account. The influence of detection anomaly results for system CM will be evaluated. Finally, the recovery of anomalous data will be considered. The false-alarm produced by CM and the performance on the practical system will also be carried out. FNR and ACC will be utilized for the data sets that include anomalous data and validate the effectiveness of sensor selection on data anomaly detection.

## Figures and Tables

**Table 1:** Description of sensor signals [33].

Index	Symbol	Description	Units
1	T2	Total temperature at fan inlet	${}^{\circ }$R
2	T24	Total temperature at LPC outlet	${}^{\circ }$R
3	T30	Total temperature at HPC outlet	${}^{\circ }$R
4	T50	Total temperature at LPT outlet	${}^{\circ }$R
5	P2	Pressure at fan inlet	psia
6	P15	Total pressure in bypass-duct	psia
7	P30	Total pressure at HPC outlet	psia
8	Nf	Physical fan speed	rpm
9	Nc	Physical core speed	rpm
10	Epr	Engine Pressure ratio	-
11	Ps30	Static pressure at HPC outlet	psia
12	Phi	Ratio of fuel flow to Ps30	pps/ps
13	NRf	Corrected fan speed	rpm
14	NRc	Corrected core speed	rpm
15	BPR	Bypass ratio	-
16	farB	Burner fuel-air ratio	-
17	htBleed	Bleed enthalpy	-
18	Nf_dmd	Demanded fan speed	rpm
19	PCNfR_dmd	Demanded corrected fan speed	rpm
20	W31	HPT coolant bleed	lbm/s
21	W32	LPT coolant bleed	lbm/s

${}^{\circ }$R: The Ranking temperature scale; psia: Pounds per square inch absolute; rpm: Revolutions per minute; pps: Pulse per second; psi: Pounds per square inch; lbm/s: Pound mass per second.

**Table 2:** Sensor data set description.

	Set 1	Set 2	Set 3	Set 4
Failure mode	1	1	2	2
Operation condition	1	6	1	6
Training units	100	260	100	248
Testing units	100	259	100	249

**Table 3:** Sensor data set for evaluation experiments.

Cycle	Sensor 2	Sensor 3	Sensor 4	Sensor 7	Sensor 8	⋯	Sensor 21
1	641.82	1589.7	1400.6	554.36	2388.1	⋯	23.4190
2	642.15	1591.8	1403.1	553.75	2388.0	⋯	23.4236
3	642.35	1588.0	1404.2	554.26	2388.1	⋯	23.3442
⋯	⋯	⋯	⋯	⋯	⋯	⋯	⋯
192	643.54	1601.4	1427.2	551.25	2388.3	⋯	22.9649

**Table 4:** Mutual information values among sensors selected by quantitative strategy for data set 1.

	Sensor 3	Sensor 4	Sensor 8	Sensor 9	Sensor 14	Sensor 15	Sensor 17
**Sensor 3**	4.6740	4.0270	2.5950	4.0353	3.9717	4.1129	1.3478
**Sensor 4**	4.0270	4.6104	2.5964	3.9717	3.9009	4.0421	1.3529
**Sensor 8**	2.5950	2.5964	3.1496	2.5614	2.4906	2.6246	0.7188
**Sensor 9**	4.0353	3.9717	2.5614	4.6187	3.9092	4.0576	1.2321
**Sensor 14**	3.9717	3.9009	2.4906	3.9092	4.5479	3.9896	1.2506
**Sensor 15**	4.1129	4.0421	2.6246	4.0576	3.9896	4.6820	1.3368
**Sensor 17**	1.3478	1.3529	0.7188	1.2321	1.2506	1.3368	1.7409

**Table 5:** Mutual information values among sensors selected by observing strategy for data set 1.

	Sensor 2	Sensor 4	Sensor 7	Sensor 8	Sensor 11	Sensor 12	Sensor 15
**Sensor 2**	4.6805	4.0335	4.0617	2.6015	3.6152	4.0455	4.1050
**Sensor 4**	4.0335	4.6104	3.9916	2.5964	3.5523	3.9681	4.0421
**Sensor 7**	4.0617	3.9916	4.6314	2.5984	3.5733	3.9891	4.0631
**Sensor 8**	2.6015	2.5964	2.5984	3.1496	2.2341	2.5822	2.6246
**Sensor 11**	3.6152	3.5523	3.5733	2.2341	4.1849	3.5643	3.6238
**Sensor 12**	4.0455	3.9681	3.9891	2.5822	3.5643	4.6152	4.0541
**Sensor 15**	4.1050	4.0421	4.0631	2.6246	3.6238	4.0541	4.6820

**Table 6:** Mutual information values among sensors selected by quantitative strategy for data set 2.

	Sensor 3	Sensor 4	Sensor 8	Sensor 9	Sensor 14	Sensor 15	Sensor 17
**Sensor 3**	4.6683	3.9870	2.2048	3.8279	3.8155	4.2054	1.4488
**Sensor 4**	3.9870	4.5060	2.0657	3.6655	3.6687	4.0508	1.3359
**Sensor 8**	2.2048	2.0657	2.6745	1.9453	1.9717	2.2503	0.4851
**Sensor 9**	3.8279	3.6655	1.9453	4.3314	3.5406	3.8685	1.2582
**Sensor 14**	3.8155	3.6687	1.9717	3.5406	4.3346	3.8794	1.2694
**Sensor 15**	4.2054	4.0508	2.2503	3.8685	3.8794	4.7167	1.4526
**Sensor 17**	1.4488	1.3359	0.4851	1.2582	1.2694	1.4526	1.7994

**Table 7:** Mutual information values among sensors selected by observing strategy for data set 2.

	Sensor 2	Sensor 4	Sensor 7	Sensor 8	Sensor 11	Sensor 12	Sensor 15
**Sensor 2**	4.6021	3.9207	3.9243	2.1695	3.5601	3.8305	4.1469
**Sensor 4**	3.9207	4.5060	3.8282	2.0657	3.4486	3.7344	4.0508
**Sensor 7**	3.9243	3.8282	4.5019	2.1235	3.4599	3.7380	4.0389
**Sensor 8**	2.1695	2.0657	2.1235	2.6745	1.7284	1.9958	2.2503
**Sensor 11**	3.5601	3.4486	3.4599	1.7284	4.1300	3.3584	3.6748
**Sensor 12**	3.8305	3.7344	3.7380	1.9958	3.3584	4.4080	3.9451
**Sensor 15**	4.1469	4.0508	4.0389	2.2503	3.6748	3.9451	4.7167

**Table 8:** Mutual information values among sensors selected by quantitative strategy for data set 3.

	Sensor 3	Sensor 4	Sensor 8	Sensor 9	Sensor 14	Sensor 15	Sensor 17
**Sensor 3**	4.6506	4.0666	2.4369	4.1106	3.9275	3.9711	1.2757
**Sensor 4**	4.0666	4.6255	2.3559	4.0855	3.8872	3.9460	1.2895
**Sensor 8**	2.4369	2.3559	2.9247	2.4529	2.2395	2.2755	0.4737
**Sensor 9**	4.1106	4.0855	2.4529	4.6694	3.9312	3.9672	1.3126
**Sensor 14**	3.9275	3.8872	2.2395	3.9312	4.4712	3.7917	1.1760
**Sensor 15**	3.9711	3.9460	2.2755	3.9672	3.7917	4.5072	1.2015
**Sensor 17**	1.2757	1.2895	0.4737	1.3126	1.1760	1.2015	1.6499

**Table 9:** Mutual information values among sensors selected by observing strategy for data set 3.

	Sensor 2	Sensor 4	Sensor 7	Sensor 8	Sensor 11	Sensor 12	Sensor 15
**Sensor 2**	4.5399	3.9559	3.8640	2.3338	3.4350	3.9346	3.8452
**Sensor 4**	3.9559	4.6255	3.9571	2.3559	3.5129	4.0201	3.9460
**Sensor 7**	3.8640	3.9571	4.5336	2.2639	3.4286	3.9207	3.8389
**Sensor 8**	2.3338	2.3559	2.2639	2.9247	1.9770	2.3781	2.2755
**Sensor 11**	3.4350	3.5129	3.4286	1.9770	4.0818	3.4689	3.4174
**Sensor 12**	3.9346	4.0201	3.9207	2.3781	3.4689	4.5965	3.9019
**Sensor 15**	3.8452	3.9460	3.8389	2.2755	3.4174	3.9019	4.5072

**Table 10:** Mutual information values among sensors selected by quantitative strategy for data set 4.

	Sensor 3	Sensor 4	Sensor 8	Sensor 9	Sensor 14	Sensor 15	Sensor 17
**Sensor 3**	4.5710	3.8083	2.3952	3.8459	3.8228	3.8139	1.2177
**Sensor 4**	3.8083	4.6736	2.4618	3.9545	3.9435	3.9285	1.2791
**Sensor 8**	2.3952	2.4618	3.1526	2.4814	2.4460	2.4675	0.6542
**Sensor 9**	3.8459	3.9545	2.4814	4.7112	3.9571	3.9541	1.2905
**Sensor 14**	3.8228	3.9435	2.446	3.9571	4.6882	3.9311	1.2809
**Sensor 15**	3.8139	3.9285	2.4675	3.9541	3.9311	4.6793	1.3216
**Sensor 17**	1.2177	1.2791	0.6542	1.2905	1.2809	1.3216	1.8265

**Table 11:** Mutual information values among sensors selected by observing strategy for data set 4.

	Sensor 2	Sensor 4	Sensor 7	Sensor 8	Sensor 11	Sensor 12	Sensor 15
**Sensor 2**	4.6695	3.9008	3.9241	2.4397	3.6265	3.8682	3.9064
**Sensor 4**	3.9008	4.6736	3.9342	2.4618	3.5946	3.8783	3.9285
**Sensor 7**	3.9241	3.9342	4.6850	2.457	3.6180	3.8776	3.9278
**Sensor 8**	2.4397	2.4618	2.457	3.1526	2.168	2.4863	2.4675
**Sensor 11**	3.6265	3.5946	3.6180	2.168	4.3634	3.5620	3.6123
**Sensor 12**	3.8682	3.8783	3.8776	2.4863	3.5620	4.6230	3.8659
**Sensor 15**	3.9064	3.9285	3.9278	2.4675	3.6123	3.8659	4.6793

**Table 12:** Mean and standard deviation of three selection strategies.

	Random Selection	Observing Selection	Quantitative Selection
**Mean**	40.93%	9.55%	3.43%
**Standard deviation**	13.68%	2.95%	2.91%
